# Tuina combined with physical therapy for spasticity of poststroke: A protocol for systematic review and meta-analysis

**DOI:** 10.1097/MD.0000000000028780

**Published:** 2022-02-11

**Authors:** Yuanyuan Ji, Yufeng Wang, Huijuan Lou, Yuxin Zhang, Yangshengjie Liu, Xu Zheng, Xiushuang Jia, Kang Yang, Hongshi Zhang, Deyu Cong

**Affiliations:** a Department of Acupuncture and Tuina, Changchun University of Chinese Medicine, Changchun, China; b Department of Tuina, Traditional Chinese Medicine Hospital of Jilin Province, China; c Department of Biochemistry and Molecular Biology, Traditional Chinese Medicine Hospital of Jilin Province, China; d Department of Ophthalmology and Otolaryngology of Chinese Medicine, Traditional Chinese Medicine Hospital of Jilin Province, Changchun, China; e Department of Nursing, Changchun University of Chinese Medicine, Changchun, China.

**Keywords:** meta-analysis, physical therapy (PT), protocol, spasticity, stroke, systematic review, Tuina

## Abstract

**Background:**

Limb spasms are a common complication of stroke. It not only affects the quality of life of stroke survivors, but also brings an economic burden. Tuina combined with physical therapy is widely used in the rehabilitation of poststroke spasticity. However, there is no supporting evidence for its efficacy and safety. This study aimed to evaluate the effectiveness and safety of Tuinas combined with physical therapy in the treatment of spasticity after stroke.

**Methods:**

Literature will be collected from the following databases: China Biology Medicine (CBM), Wanfang Database, China National Knowledge Infrastructure (CNKI), Chinese Scientific Journal Database (VIP), PubMed, Embase, Cochrane Library, and Web of Science; We will include randomized controlled trials of Tuina combined with physical therapy for poststroke spasticity range from the establishment to May 1, 2021. There were no limitations to the publication time, and the language was limited to Chinese and English. The primary outcome was evaluated using the Modified Ashworth scale, and the secondary outcomes were the simplified Fugl-Meyer Assessment scale, Modified Barthel Index, Functional Independence Measurement (FIM), and Visual Analog Scale. RevMan V.5.4.1 software was used for the meta-analysis. The Cochrane Intervention System Evaluation Manual analyzes the risk of bias, and the recommended grading assessment, development and evaluation are used to assess the quality of evidence.

**Ethics and dissemination:**

This study will be based on published systematic review studies, no ethical approval is required and the results of the study will be published in a peer-reviewed scientific journal.

**Systematic review registration:**

INPLASY2021110064.

## 1 Introduction

Stroke is a leading cause of death and long-term disability worldwide.^[1]^ The incidence of stroke and its subcategories increased in China from 1990 to 2019, with ischemic stroke increasing the most, followed by subarachnoid and intracerebral hemorrhage.^[2]^ Limb spasm is a common complication after stroke, occurring in 30% to 80% of stroke survivors.^[3]^ Spasticity following stroke often interacts with pain, weakness, soft tissue stiffness, and joint contracture, resulting in disordered motor control and functional limitations, reduced quality of life, and an increased burden of treatment costs and caregivers.^[3–5]^

The treatment goals of spastic disorder after stroke are to promote the recovery of limb motor function and correct abnormal posture to improve the independent survival ability of patients and reduce the patients’ care burden.^[6]^ The recovery of motor function is a complex process that depends not only on muscle tone but also on strength, endurance, sensation, and coordination.^[7]^ Based on the complexity of poststroke spasticity, multidisciplinary comprehensive therapies are more effective than pharmacological treatment alone,^[7–9]^ however, the cost burden on patients should also be considered. A United Kingdom study found that the cost of limb spasms followed by stroke is as high as that without spasm.^[10]^ Therefore, combining rehabilitation technology with cost-effective medical treatment may provide the most beneficial outcomes for patients with poststroke spasms. Tuina, a key component of traditional Chinese medicine, is a passive mechanical therapy and cost-effective adjuvant therapy.^[11]^ Systematic evaluation shows that therapeutic massage, especially Tuina massage, is effective in improving motor function and reducing spasm in stroke survivors.^[12]^ In this article, physical therapy refers to the method of exercise training with bare hands or the application of equipment to restore or improve dysfunction after stroke. Physical therapy (PT) is a key treatment method for stroke rehabilitation, usually in the early stages and continuing into the chronic stage poststroke, aimed at recovering and maintaining activities of daily living (ADL).^[13]^ Studies have shown that PT intervention is beneficial for highly repetitive task orientation and task-specific training at all stages poststroke.^[14]^ In clinical practice, Tuina combined with PT is a widely used approach for poststroke spasticity; however, there is no scientific evidence. Therefore, this systematic review aimed to assess the effectiveness and safety of Tuina combined with PT for limb spasms after stroke and to provide better clinical decision-making.

## 2 Methods and analysis

The systematic review was based on the guidelines of the Preferred Reporting Items for Systematic Review and Meta-analysis Protocol (PRISMA-P).^[15]^ The protocol for this study was registered in the INPLASY. (ID: INPLASY2021110064).

### 2.1 Inclusion criteria

#### 2.1.1 Types of study

We will only include randomized controlled clinical trials of Tuina combined with physical therapy for limb spasm after stroke without limitation of publication status, and the language will be limited to Chinese and English.

#### 2.1.2 Types of participants

All patients must meet the WHO diagnostic criteria for stroke^[16]^ and be over 18 years old. Participants had symptoms of increased muscle tone in their limbs, with spasm rated as 1 to 2 according to the modified Ashworth scale. Not accompanied by other diseases that cause limb spasms. The vital signs are stable, patients with serious primary diseases such as liver, kidney, hematopoietic system and endocrine system, visual and auditory impairment, severe cognitive impairment, mental illness, who cannot cooperate with examination and rehabilitation will be excluded. There were no restrictions on gender, race, or country.

#### 2.1.3 Types of interventions

The intervention method for the treatment group was Tuina combined with PT. In this study, the PT method mainly refers to kinesiotherapy, excluding physical factor therapy. The control group received routine rehabilitation treatments, such as medication, physical therapy, Botox injection, and acupuncture etc.

#### 2.1.4 Types of outcome measures

##### 2.1.4.1 Main outcome

The primary outcome will be evaluated by the Modified Ashworth scale, which is commonly used to assess the severity of spasmodic hypertonia in clinical.^[17,18]^

##### 2.1.4.2 Secondary outcome

A simplified Fugl-Meyer motor assessment was used to assess motor function. The visual analog scale was used for the assessment of limb pain. Activities of daily living were measured using the modified Barthel index. Functional independence measurement (FIM) was used to evaluate the independence level of stroke rehabilitation patients.

### 2.2 Exclusion criteria

- Case reports, reviews, observational studies, animal experiments, quasi-randomized controlled trials.
- The full text of the articles is not available or data is incomplete.
- Duplicate published studies.

### 2.3 Search methods

#### 2.3.1 Electronic searches

We will search for articles from the China Biology Medicine (CBM), Wanfang Database, China National Knowledge Infrastructure (CNKI), Chinese Scientific Journal Database (VIP), PubMed, Embase, Cochrane Library, and Web of Science. We will also consult the bibliography, and the articles will be searched from their inception to May 1, 2021. The language of the articles was Chinese and English. The specific search strategy for PubMed is presented in Table 1.

**Table 1:** Search strategy of PubMed.

No	Search terms
#1	Tuina (all fields)
#2	Massage (all fields)
#3	Or #1-#2
#4	Stroke (all fields)
#5	Poststroke (all fields)
#6	After stroke (all fields)
#7	Ischemic stroke (all fields)
#8	Apoplectic (all fields)
#9	Cerebrovascular accident (all fields)
#10	Cerebrovascular disorder (all fields)
#11	Apoplexy (all fields)
#12	Cerebral hemorrhage (all fields)
#13	OR #4-#12
#14	Spasticity (all fields)
#15	Spasm (all fields)
#16	Muscle spasticity (all fields)
#17	Spastic hemiplegia (all fields)
#18	Limb spasm (all fields)
#19	High tone (all fields)
#20	OR #14-#19
#21	Randomized controlled trial (all fields)
#22	Controlled clinical trial (all fields)
#23	Randomized (all fields)
#24	Randomly (all fields)
#25	Or#21-#24
#26	#3 and #13 and #20 and #25

### 2.4 Data collection and analysis

#### 2.4.1 Selection of study

**Figure 1:**
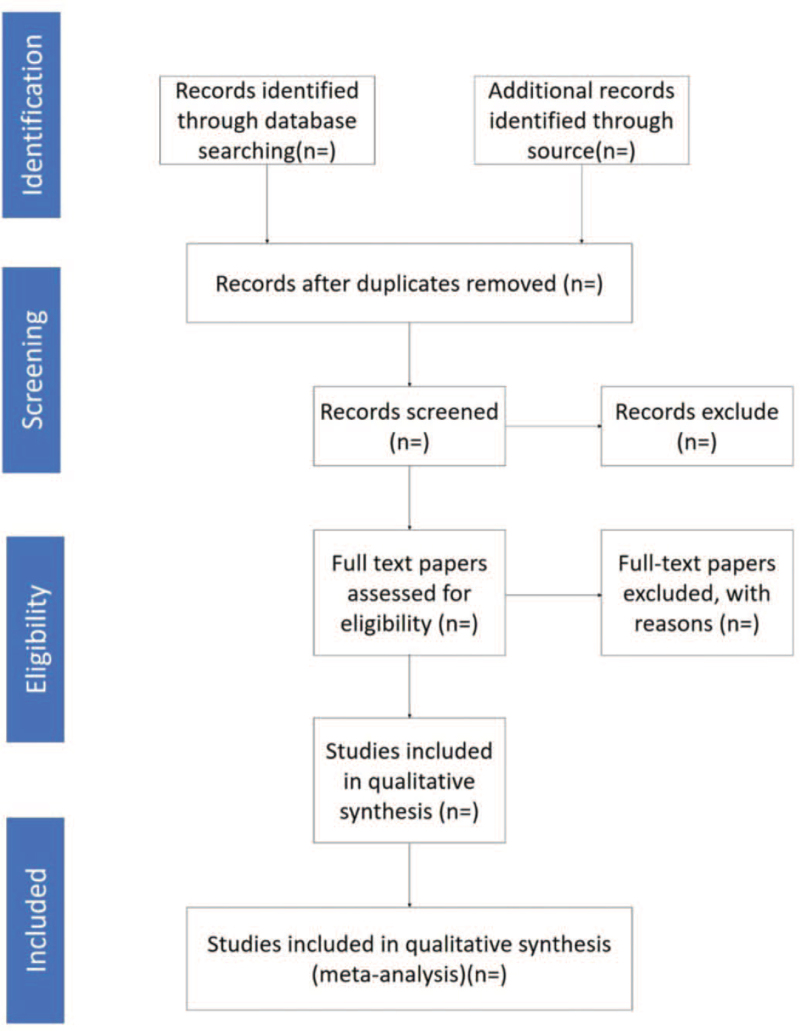
Flow diagram of study selection process.

All the retrieved articles were managed using EndNote X9. Two researchers screened the literature according to the inclusion and exclusion criteria independently after reading the title and abstract, then remaining the eligible articles and deleting the texts that did not meet the inclusion criteria. Subsequently, further confirmation was obtained by reading the full text. If 2 researchers disagree about the inclusion of the paper, the third researcher will make the judgment. The filtering process is illustrated in Figure 1.

#### 2.4.2 Data extraction

Data were independently extracted from the included studies by 2 researchers. The extracted data included the first author, publication year, sample size, method of treatment, outcome indicators, and adverse events. If the information in some articles is incomplete, the author will be contacted to ensure the accuracy of the information.

### 2.5 Assessment of risk of bias

Two researchers will use the Cochrane Collaboration bias risk assessment tool to assess the risk of bias independently. The risks in the following areas of deviation will be assessed: random sequence generation, assignment concealment, blinded subjects and therapists, blinded evaluators, incomplete outcome data, selective outcome reports, and other deviations. The evaluation results were divided into low, high, and uncertain risks. Disagreements were resolved by discussion with a third researcher.

### 2.6 Assessment of heterogeneity

Heterogeneity was tested for all the included studies using *I*^2^ statistics. There was no obvious heterogeneity when the *I*^2^ value was <50%. Otherwise, if the result of *I*^2^ was greater than 50%, we considered that there was obvious heterogeneity and conducted subgroup and sensitivity analyses to explore the source of heterogeneity.

### 2.7 Assessment of reporting bias

If more than 10 trials met the study criteria, funnel plots were drawn and analyzed using Review Manager software (version 5.3), and potential publication bias was assessed using funnel plots.

### 2.8 Data synthesis

Review Manager 5.3 provided by the Cochrane Collaborative Network will be used for the meta-analysis. Dichotomous results will be analyzed using relative risk, and continuous results will be analyzed using mean difference or standardized mean difference with a 95% confidence interval. Based on the heterogeneity, we choose different effect models. When the statistical heterogeneity was low (*P* > .1, or *I*^2^ < 50), we used the fixed-effects model to combine the data; otherwise, a random effects model was used. However, if this cannot be performed, we will conduct a descriptive analysis.

### 2.9 Subgroup analysis

To assess the heterogeneity of this study, we performed a subgroup analysis based on the following factors: sex, age, type of stroke, type of Tuina or physical therapy, spasm parts, control group, time of treatment, and course of treatment.

### 2.10 Sensitivity analysis

A sensitivity analysis was performed to assess the robustness and reliability of the pooled results. Studies with high risk of bias were excluded. The meta-analysis will be repeated, when significant statistical heterogeneity is present to assess the quality and robustness based on sample size and insufficient data.

### 2.11 Grading the quality of evidence

In order to evaluate the quality of evidence, we used the Grading of Recommendations Assessment, Development, and Evaluation^[19]^ system. The evaluation mainly included the following aspects: heterogeneity, imprecision, publication bias, indirectness, and risk of bias. The outcomes are ranked as “high”, “moderate”, “low”, and “very low”.

### 2.12 Ethics and dissemination

As this study will be based on published systematic review studies, no ethical approval is required and the results of the study will be published in a peer-reviewed scientific journal in accordance with the PRISMA guidelines.

## 3 Discussion

Spasticity is the main motor disorder after stroke, and poses major challenges to treatment and patient care.^[20]^ Therefore, there is an urgent need to develop cost-effective treatments. Tuina combined with physical therapy can effectively reduce limb spasms and improve motor function in patients after stroke. TCM Tuina is a passive therapy with a good therapeutic effect on skeletal muscle lesions. Therapeutic massage (Tuina) is thought to increase muscle mass, blood flow, and muscle tissue temperature, which may help increase muscle compliance and reduce muscle stiffness.^[21,22]^ In addition, several studies^[23,24]^ have shown that tactile stimulation is beneficial to restore sensorimotor function after stroke. Sen et al^[25]^ demonstrated that mechanical massage improves sensorimotor behavior after stroke, maintains gait, and reduces inflammation and the subacute expression of metabolic muscle factors. Massage in various settings has been shown to have therapeutic benefits for motor and neuromuscular systems.^[26]^ Therefore, it is reasonable to conclude that the Tuina can effectively improve limb spasms and motor dysfunction after stroke. PT is an active therapy typically used for the treatment of motor dysfunction. The combination of passive and active therapies plays a role in improving the treatment effect and maximizing self-efficacy.^[27]^ There is still a lack of high-quality evidence to demonstrating that Tuina combined with PT is effective in treating poststroke spasticity. Therefore, this study aimed to assess the effectiveness and safety of Tuinas combined with PT, using a systematic review and meta-analysis of randomized controlled trial data. We hope that the results of this study will help establish a better treatment for poststroke spasm and provide reliable evidence for its wide application.

## Author contributions

**Data curation:** Huijuan Lou, Yuxin Zhang.

**Formal analysis:** Yangshengjie Liu. Xiushuang Jia.

**Funding acquisition:** Deyu Cong.

**Investigation:** Hongshi Zhang, Yuxin Zhang.

**Methodology:** Kang Yang, Xu Zheng.

**Validation:** Yufeng Wang, Yuanyuan Ji.

**Writing – original draft:** Yuanyuan Ji.

**Writing – review & editing:** Deyu Cong. Yuanyuan Ji.

## References

[1] Caprio FZ Sorond FA. Cerebrovascular disease: primary and secondary stroke prevention. Med Clin North Am 2019;103:295–308. 30704682 10.1016/j.mcna.2018.10.001

[2] Ma Q Li R Wang L. Temporal trend and attributable risk factors of stroke burden in China, 1990–2019: an analysis for the Global Burden of Disease Study 2019. Lancet Public Health 2021;6:e897–906. 34838196 10.1016/S2468-2667(21)00228-0 PMC9047702

[3] Kuo C-L Hu G-C. Post-stroke spasticity: a review of epidemiology, pathophysiology, and treatments. Int J Gerontol 2018;12:280–4.

[4] Li S Chen YT Francisco GE Zhou P Rymer WZ. A unifying pathophysiological account for post-stroke spasticity and disordered motor control. Front Neurol 2019;10:468. 31133971 10.3389/fneur.2019.00468 PMC6524557

[5] Schinwelski MJ Sitek EJ Wąż P Sławek JW. Prevalence and predictors of post-stroke spasticity and its impact on daily living and quality of life. Neurol Neurochir Pol 2019;53:449–57. 31845749 10.5603/PJNNS.a2019.0067

[6] Sunnerhagen K Olver J Francisco G. Assessing and treating functional impairment in poststroke spasticity. Neurology 2013;80:S35–44. 23319484 10.1212/WNL.0b013e3182764aa2

[7] Francisco G McGuire J. Poststroke spasticity management. Stroke 2012;43:3132–6. 22984012 10.1161/STROKEAHA.111.639831

[8] Smania N Picelli A Munari D. Rehabilitation procedures in the management of spasticity. Eur J Phys Rehabil Med 2010;46:423–38. 20927008

[9] Bethoux F. Spasticity management after stroke. Phys Med Rehabil Clin N Am 2015;26:625–39. 26522902 10.1016/j.pmr.2015.07.003

[10] Raluy-Callado M Cox A MacLachlan S. A retrospective study to assess resource utilization and costs in patients with post-stroke spasticity in the United Kingdom. Curr Med Res Opin 2018;34:1317–24. 29490512 10.1080/03007995.2018.1447449

[11] Barnes PM Bloom B Nahin RL. Complementary and alternative medicine use among adults and children: United States, 2007. Natl Health Stat Report 2008;10:01–23. 19361005

[12] Cabanas-Valdés R Calvo-Sanz J Serra-Llobet P Alcoba-Kait J González-Rueda V Rodríguez-Rubio PR. The Effectiveness of Massage Therapy for Improving Sequelae in Post-Stroke Survivors. A Systematic Review and Meta-Analysis. Int J Environ Res Public Health 2021;18:4424. 33919371 10.3390/ijerph18094424 PMC8122530

[13] Langhorne P Bernhardt J Kwakkel G. Stroke rehabilitation. Lancet 2011;377:1693–702. 21571152 10.1016/S0140-6736(11)60325-5

[14] Veerbeek JM van Wegen E van Peppen R. What is the evidence for physical therapy poststroke? A systematic review and meta-analysis. PLoS One 2014;9:e87987. 24505342 10.1371/journal.pone.0087987 PMC3913786

[15] Moher D Shamseer L Clarke M. Preferred reporting items for systematic review and meta-analysis protocols (PRISMA-P) 2015 statement. Syst Rev 2015;4:01. 10.1186/2046-4053-4-1 PMC4320440 25554246

[16] Liu Z Guan L Wang Y Xie C Lin X Zheng G. History and mechanism for treatment of intracerebral hemorrhage with scalp acupuncture. Evid Based Complement Alternat Med 2012;2012:895032. 22474527 10.1155/2012/895032 PMC3296221

[17] Gregson J Leathley M Moore A Sharma A Smith T Watkins C. Reliability of the tone assessment scale and the modified Ashworth scale as clinical tools for assessing poststroke spasticity. Arch Phys Med Rehabil 1999;80:1013–6. 10489001 10.1016/s0003-9993(99)90053-9

[18] Alibiglou L Rymer WZ Harvey RL Mirbagheri MM. The relation between Ashworth scores and neuromechanical measurements of spasticity following stroke. J Neuroeng Rehabil 2008;5:18. 18627628 10.1186/1743-0003-5-18 PMC2515334

[19] Langer G Meerpohl JJ Perleth M Gartlehner G Schünemann H. GRADE guidelines: 12. Developing Summary of Findings tables - dichotomous outcomes. Z Evid Fortbild Qual Gesundhwes 2013;107:646–64. 24315336 10.1016/j.zefq.2013.10.034

[20] Li S. Spasticity, motor recovery, and neural plasticity after stroke. Front Neurol 2017;8:120. 28421032 10.3389/fneur.2017.00120 PMC5377239

[21] Qais G Wurida S. Determining the benefits of massage mechanisms: a review of literature. Rehabil Sci 2017;2:58–67.

[22] Drust B Atkinson G Gregson W French D Binningsley D. The effects of massage on intra muscular temperature in the vastus lateralis in humans. Int J Sports Med 2003;24:395–9. 12905085 10.1055/s-2003-41182

[23] Gibb RL Gonzalez CL Wegenast W Kolb BE. Tactile stimulation promotes motor recovery following cortical injury in adult rats. Behav Brain Res 2010;214:102–7. 20394780 10.1016/j.bbr.2010.04.008

[24] Lämås K Häger C Lindgren L Wester P Brulin C. Does touch massage facilitate recovery after stroke? A study protocol of a randomized controlled trial. BMC Complement and Altern Med 2016;16:50. 26846253 10.1186/s12906-016-1029-9 PMC4743203

[25] Sen CK Khanna S Harris H. Robot-assisted mechanical therapy attenuates stroke-induced limb skeletal muscle injury. FASEB J 2017;31:927–36. 27895105 10.1096/fj.201600437R PMC5295731

[26] Balch MHH Harris H Chugh D. Ischemic stroke-induced polyaxonal innervation at the neuromuscular junction is attenuated by robot-assisted mechanical therapy. Exp Neurol 2021;343:113767. 34044000 10.1016/j.expneurol.2021.113767 PMC8286354

[27] Eubanks JE, Chang Chien GC, Atchison JW. Manipulation, Mobilization, Massage and Traction in Pain Management. In: Abd-Elsayed A. (eds) Pain. Springer, Cham. 2019 10.1007/978-3-319-99124-5_223.

